# Electroencephalography in young onset dementia

**DOI:** 10.1186/s12883-023-03248-w

**Published:** 2023-05-23

**Authors:** Casey W Brown, Huei-Yang Chen, Peter K Panegyres

**Affiliations:** 1 Faculty of Health and Medical Sciences, The University of Western Australia, Crawley, 6009 Australia; 2 Neurodegenerative Disorders Research Pty Ltd, 4 Lawrence Avenue, West Perth, West Perth, WA 6005 Australia

**Keywords:** Young onset dementia, Electroencephalography, Alzheimer’s disease: frontotemporal dementia

## Abstract

**Background:**

Young onset dementia (YOD) is a major diagnostic and management problem.

**Methods:**

We set out to explore if electroencephalography (EEG) might be useful in the diagnosis of young onset Alzheimer’s disease (YOAD) and young onset frontotemporal dementia (YOFTD). The ARTEMIS project is a 25-year prospective study of YOD based in Perth, Western Australia. 231 participants were included: YOAD: n = 103, YOFTD: n = 28, controls: n = 100. EEGs were performed prospectively, with 30-minute recording time for each subject, without knowledge of diagnosis or other diagnostic data.

**Results:**

80.9% of patients with YOD had abnormal EEGs (*P* < 0.00001). Slow wave changes were more frequent in YOAD that YOFTD (*P* < 0.00001), but no difference in the frequency of epileptiform activity (*P* = 0.32), with 38.8% of YOAD and 28.6% of YOFTD patients having epileptiform activity. Slow wave changes were more generalized in YOAD (*P* = 0.001). Slow wave changes and epileptiform activity were not sensitive to the diagnosis of YOD, but highly specific (97–99%). The absence of slow wave changes and epileptiform activity had a 100% negative predictive value and likelihood radio 0.14 and 0.62 respectively, meaning that those without slow wave changes or epileptiform activity had low probability of having YOD. No relationship was established between EEG findings and the patient’s presenting problem. Eleven patients with YOAD developed seizures during the study, and only one with YOFTD.

**Conclusions:**

The EEG is highly specific for the diagnosis of YOD with the absence of slow wave changes and epileptiform phenomena making the diagnosis unlikely, with 100% negative predictive value and with low probability for the dementia diagnosis.

## Background

Dementia is a common syndrome which is characterised by a decline in functioning secondary to impaired cognition. There are many subtypes of dementia which differ in the pathophysiology that underlies the condition and in the typical presentation. The most common subtypes of dementia are Alzheimer’s disease, frontotemporal dementia, vascular dementia, and Lewy body dementia [1]. Although dementia is typically a disease of the older population, there is a proportion of patients in which the onset of the disorder is prior to the age of 65. This is referred to as young onset dementia (YOD) [2–6].

The burden on people with young onset dementia and their families is immense, with large financial, social and emotional aspects [7]. An early diagnosis of dementia is important as it provides patients, their families and their carers access to important health care and other services which may help to lessen this significant burden [5]. Furthermore, as young onset dementia is more likely to have a treatable cause as opposed to late onset dementia [8], an early diagnosis is key as it allows patients to access new treatments as they become available before irreversible damage has occurred [9].

To allow for early diagnosis of young onset dementia, reliable diagnostic biomarkers are required. Some biomarkers currently exist, including computerised tomography, magnetic resonance imaging, specialised cerebrospinal fluid (CSF) analyses and positron emission tomography (PET) with fluorodeoxyglucose. However, all of these biomarkers are costly, and many are hard to access outside of highly specialised centres. Hence a low-cost and easily accessible biomarker is required.

One potential biomarker for young onset dementia is electroencephalography (EEG). EEG is a low-cost investigation of brain dynamics which provides excellent temporal resolution [10]. Current research on EEG as a biomarker of dementia does show characteristic changes. A frequent abnormality in Alzheimer’s disease is an increase in theta and delta activity, known as slow wave changes, and a decrease in alpha and beta activity [11–14]. Furthermore, an increase in slow wave change severity has been associated with worse outcomes such as more severe cognitive impairment and psychosis [15, 16]. This increase may also be associated with clinical progression from mild cognitive impairment to dementia [17]. Another well reported EEG abnormality is the presence of epileptiform activity [18, 19], which may be triggered by beta-amyloid deposits [20]. Much of the current research uses spectral analysis EEG as a biomarker [21–23]. However, this form of EEG is a large expensive highly specialised piece of equipment. Hence this paper will focus on plain EEG, as it is far more accessible for most populations worldwide because of its low cost.

While there are well reported EEG abnormalities in dementia, there are few studies of YOD [24–26]. Previous studies have shown that patients with young onset Alzheimer’s disease (YOAD) have slow wave changes on EEG, and that these slow wave changes are more severe than in late onset Alzheimer’s disease [14, 27, 28]. This correlation between abnormality severity and age was the opposite for healthy controls, suggesting that it is not a result of normal aging. Studies have also shown that young onset frontotemporal dementia (YOFTD) patients also have slow wave changes on EEG [29, 30]. However, there is no consensus on whether there is significant difference between EEG abnormalities in YOAD and YOFTD. These studies, however, were retrospective, and there is a need for studies with improved design.

The current study aims to investigate the EEG as a diagnostic biomarker in two of the most common young onset dementia subtypes—YOAD and YOFTD—using a longitudinal prospective design. It is hypothesised that EEG abnormalities will be more prevalent in patients with YOAD and YOFTD compared to healthy controls. As this paper is looking at a potential diagnostic biomarker, the EEG abnormalities will be compared to the presenting problem for each patient in an attempt to find clinical correlates. Furthermore, as it is known that dementia patients are more likely to develop seizures and epilepsy [31–33], this study will also report the development of clinical seizure activity in its participants.

## Methods

This is a 25-year longitudinal prospective cohort analysis of patients—who were referred by general practitioners, geriatricians, psychologists and neurologists—with the possible diagnosis of YOD: known as the ARTEMIS Project (JHC HREC: ARTEMIS 1406). Young onset dementia is defined as dementia with an onset prior to 65 years of age [4–6]. The ARTEMIS Project involves the assessment of these patients in community-based clinics—established by one of the authors—specialising in neurodegenerative disorders in young adults. These patients, their carers and their families were seen at least six-monthly for a median of 10 years (3–20 years).

Diagnoses were made at the time of enrolment in the project using published criteria, and each patient had their diagnosis reviewed at each clinic visit using the most up-to-date diagnostic criteria. The diagnosis of dementia was based on a decrease in functioning secondary to cognitive decline or behavioural changes which were not otherwise explained by delirium or psychiatric disease. Dementia was diagnosed using the concepts of major cognitive disorder [34]. Patients with dementia had decline in acts of daily living, as measured by the Lawton Instrumental Activities of Daily Living Scale (IADL) and all dementia patients scored ≤ 3, indicating significant functional decline [35, 36]. Cognitive decline was diagnosed through history taking and the use of cognitive assessments: including Addenbrooke’s Cognitive Examination – Revised 2005 (ACE-R), Total Function Capacity (TFC), Mini-Mental State Exam (MMSE),, and Depression, Anxiety and Stress Scores (DASS).

Alzheimer’s disease was diagnosed using the original 1984 guidelines from the National Institute of Neurological and Communicative Disorders and Stroke (NINCDS) [37], and the revision of these guidelines by the Alzheimer’s Disease and Related Disorders Association (ADRDA) [38]. All patients with YOAD were diagnosed using the revised NINCDS-ADRDA criteria with all patients having at least one biomarker of structural imaging with MRI and molecular imaging with FDG-PET scanning [38]. Frontotemporal dementia was diagnosed using a variety of published criteria which evolved over time [39–41]. All patients had MRI and brain FDG-PET to secure the diagnosis of YOAD or YOFTD. Amyloid PET scanning was performed in 2 AD patients where there was uncertainty as to the clinical diagnosis. No patients required CSF analysis for this study. Only patients with an amnestic presentation of YOAD or behavioural variant of YOFTD were incorporated into the study.

The present paper included participants who received a diagnosis of YOAD (*n* = 103) or a diagnosis of YOFTD (*n* = 28). The healthy age-matched controls were selected from the well spouses and partners of the patients who accompanied the patients to the clinic; the controls had no history of cognitive symptoms and no neurological history such as stroke, epilepsy or migraine.

Patients who had clinical neurological or imaging findings to suggest Lewy body disease [42, 43] or vascular dementia were excluded [44, 45]. Twenty-four patients with coexistent Lewy body disease and vascular dementia were excluded using these diagnostic criteria and all patients included in the study had a Fazekas score of 0–1 [46].

Demographic information, time from onset of dementia to EEG and cognitive data around time of EEG are summarised in Table 1. While there was a larger proportion of males in the YOFTD group (57.1% compared to 40.8% in the YOAD group and 48.0% in the control group), this difference was not statistically significant (χ^2^ 12.69, *P* = 0.26). Furthermore, there was no significant difference noted in the mean age of onset for either disease group (57.3 vs. 57.9, *P* = 0.59).

**Table 1:** Demographic data, time from onset of dementia to EEG and cognitive data

	Young onset Alzheimer’s disease	Young onset frontotemporal dementia	Controls
N	103	28	100
Median age of onset	57	57	58
Range of age of onset	45–64	41–64	30–65
Sex (%) - male	40.8	57.1	48
Time from clinical onset to EEG:			
Median (month) 25% 1QR 75% 1QR	6 6 14	7 7 8	
Cognitive tests at time of EEG:	*± SD (median)*	*± SD (median)*	
MMSE ACE-R DASS: D A S TFC CBI-R FRS	20.8 ± 6.0 (27) 59.8 ± 19.8 (64) 8.3 ± 9.7 (9) 7.6 ± 8.7 (5) 10.1 ± 8.6 (8) 9.6 ± 2.8 (10) 51.1 ± 32.2 (44) 49.9 ± 22 (54)	24.0 ± 4.8 (26) 70.0 ± 19.1 (72) 7.0 ± 6.7 (6) 5.8 ± 6.3 (5) 11.1 ± 8.2 (10) 10.9 ± 2.1 (12) 51.2 ± 30.7 (50) 47.9 ± 27.0 (46.5)	

The EEGs were recorded by the same technical staff using a Neurofax EEG-9000 series machine and were perfomed with 35 Hz filters using three montages anterior-posterior longitudinal bipolar, reference and traverse bipolar with Supersense gold cup electrodes and Everi conductive and abrasive paste to prepare the skin, and Elefix paste to affix the electrodes. Recording time was 30 min with alternating runs of eyes open (20 s) and eyes closed (40 s) for each montage. Photic stimulation was performed using the longitudinal bipolar montage at each flash frequency for 1 to 20 Hz with 4 s eyes open, then closed. All EEGs (*n* = 205) were examined blind as to the diagnoses and investigational data. EEGs were performed within 3 months of presentation to the clinics. The EEGs were reported by expert technicians and finalized after neurological review by staff with over 40 years’ collective experience in performing and reporting EEGs. Each participant’s EEG was reported individually and separately with a single report being issued. Only one neurologist reported on all the EEGs after review and discussion of each EEG with the same technical staff who performed the studies, minimizing inter-rater variability. Abnormalities included slow wave changes and epileptiform activity. Theta activity is 4–7 Hz of voltage greater than 30µV and either persistently lateralized to one hemisphere or localized to one region to indicate regional dysfunction, disrupting background rhythm and not related to drowsiness. Theta may be diffuse, with slight accentuation in left temporal region. Delta activity is 0.5 to 3 Hz waves and unrelated to drowsiness, sleep or hyperventilation. Intermittent rhythmic delta (IRDA) is 1–3 Hz in brief bursts and widely distributed, usually maximum anteriorly and may have regional or focal delta anomalies. Delta may also be diffuse bilateral and arrhythmic 0.5–3 Hz. Slow wave changes were not related to drowsiness or medication effects.

Focal spikes are apiculate wave forms distinct from background, interrupt background rhythm and have more than one phase. Largest phase electronegative, with asymmetrical scores, involve more than one electrode position and followed by a slow wave. Multifocal spikes involve > 3 spike foci with at least one in each hemisphere. Generalized spike-wave and polyspike-wave complexes are usually bilateral with repetition rate 3.5–5 Hz. Bursts of polyspikes repeating at 10–25 Hz with irregular discharge rate may be generalized but usually frontal at 40–350µV with burst duration 1–8s [47].

The specific types of abnormalities were noted; increases in theta or delta activity defined. The specific types of epileptiform activity were sharp waves, spike and waves, or sharp and slow waves. The anatomic locations of the abnormalities were recorded.

The initial case reports between the referring doctor and the clinic for each patient were also examined, and the presenting problem recorded. The presenting problem is defined as the symptom or the issue which caused the patient to seek help and be referred to the clinic, as recorded in the patients’ own words. Presenting problems were categorised into five categories: memory disturbance, behavioural changes, mood disturbance, language deficit, and motor disturbance. Memory disturbance was defined as any decrease from baseline in any type of memory, such as long-term memory or short-term memory. Behavioural changes were defined as any change in the patient’s behaviour, such as an increase in aggression, obsessionality, or inappropriate behaviour. Mood disturbance was any increase or decrease in mood that was not otherwise explained. Language deficits represented any decrease in language fluency, including problems with language production or language comprehension. Note that word finding difficulties were considered a memory disturbance and not a language deficit. Motor disturbance was any new difficulty with motor functioning that was not otherwise explained, such as paraparesis, apraxia, or dystonia.

Each patient’s case records were searched for any history of clinical seizures. For all patients with clinical seizures, note was made of the types of seizures (e.g. generalised tonic-clonic seizures) and the time of their first seizure in relation to the onset of dementia.

Statistical analysis consisted of chi-squared analysis, Fisher’s exact test, and Student’s *t*-test as appropriate. The analysis was conducted using Microsoft Excel, version 2019. Sensitivity, specificity, positive predictive values (PPV) and negative predictive values (NPV) and likelihood ratios were calculated using standard methodology [48]. The ascertainment of PPVs and NPVs used prevalence estimates as published: 41.1 per 100,000 for YOAD and 2.3 per 100,000 for YOFTD [49].

## Results

The EEG abnormalities seen in the YOAD group and YOFTD group are shown in Table 2. In the control group, only four patients (4%) had abnormal EEGs (one patient had epileptiform activity and three patients had slow wave changes). Both disease groups were significantly more likely to have abnormalities on EEG than the control group, with 91 (88.3%) of the YOAD group (χ^2^ 145.0, *P* < 0.00001) and 15 (53.6%) of the YOFTD group (χ^2^ 42.5, *P* < 0.00001). The YOAD group had significantly more EEG abnormalities than the YOFTD group (χ^2^ 17.2, *P* < 0.0001).

**Table 2:** EEG abnormalities found in patients with young onset Alzheimer’s disease or young onset frontotemporal dementia, including sensitivity, specificity, positive predictive values and negative predictive values and likelihood ratios

	Alzheimer’s disease (*n* = 103)	Frontotemporal dementia (*n* = 28)	Healthy controls (*n* = 100)
N without EEG abnormality**	12 (11.7%)	13 (46.4%)	96 (96.0%)
N with slow wave changes**	89 (86.4%)^+^	12 (42.9%)^++^	3 (3.0%)
N with epileptiform changes	40 (38.8%)^+++^	8 (28.6%)^++++^	1 (1.0%)

* *P* < 0.05

** *P* < 0.001

^+^ sensitivity 86.4%; specificity 97.0%; positive predictive value 1.10%; negative predictive value 100%; PLR = 28.8, NLR = 0.14

^++^ sensitivity 42.9%; specificity 97.0%; positive predictive value 0.03%; negative predictive value 100%; PLR = 14.3, NLR = 0.59

^+++^ sensitivity 38.8%; specificity 99.0%; positive predictive value 1.50%; negative predictive value 100%; PLR = 38.8, NLR = 0.62

^++++^ sensitivity 28.6%; specificity 99.0%; positive predictive value 0.06%; negative predictive value 100%; PLR = 28.6, NLR = 0.72

(PLR = positive likelihood ratio; NLR = negative likelihood ratio)

Considering the two disease groups, those with YOAD were significantly more likely to have slow wave changes than those with YOFTD (86.4% vs. 42.9%, χ^2^ 42.9, *P* < 0.00001), as seen in Table 2. Of those with slow wave changes, patients with YOAD were more likely than those with YOFTD to have generalised changes (47.2% vs. 0.0%, *P* = 0.001) (Table 3). Of those with slow wave changes, there were no significant differences between the two groups in prevalence of changes in specific anatomical locations (Table 3). There was also no significant difference between the two groups in terms of types of slow wave changes. There was no significant difference between rates of epileptiform activity in the two disease groups (YOAD 38.8% vs. YOFTD 28.6%, χ^2^ 1.0, *P* = 0.32) (Table 2). Of those with epileptiform activity, no significant difference was found between the two groups in terms of anatomical location or type of activity (Table 4).

**Table 3:** The type and location of slow wave changes found in patients with young onset Alzheimer’s disease and young onset frontotemporal dementia

	Alzheimer’s disease (*n* = 89)	Frontotemporal dementia (*n* = 12)
**Type of slow wave changes**		
Only theta frequencies	25 (28.1%)	6 (50.0%)
Only delta frequencies	11 (12.4%)	0 (0.0%)
Theta and delta frequencies	53 (59.6%)	6 (50.0%)
**Location of slow wave changes**		
Left frontal	72 (80.1%)	9 (75.0%)
Right frontal	62 (69.7%)	8 (66.7%)
Left temporal	70 (78.7%)	10 (83.3%)
Right temporal	57 (64.0%)	10 (83.3%)
Left posterior	10 (11.2%)	1 (8.3%)
Right posterior	9 (10.1%)	1 (8.3%)
Central	18 (20.2%)	1 (8.3%)
Generalised*	42 (47.2%)	0 (0.0%)

* *P* < 0.001

**Table 4:** The type and location of epileptiform activity found in patients with young onset Alzheimer’s disease and young onset frontotemporal dementia

	Alzheimer’s disease (*n* = 40)	Frontotemporal dementia (*n* = 8)
**Type of epileptiform activity**		
Sharp wave	22 (55.0%)	5 (62.5%)
Spike and wave	17 (42.5%)	2 (25.0%)
Sharp and slow waves	23 (57.5%)	5 (62.5%)
**Location of epileptiform activity**		
Left frontal	22 (55.0%)	2 (25.0%)
Right frontal	16 (40.0%)	3 (37.5%)
Left temporal	24 (60.0%)	3 (37.5%)
Right temporal	21 (52.5%)	4 (50.0%)
Left posterior	2 (5.0%)	1 (12.5%)
Right posterior	6 (15.0%)	1 (12.5%)
Central	11 (27.5%)	1 (12.5%)
Generalised	5 (12.5%)	0 (0.0%)

Slow wave changes and epileptiform activity had low sensitivity for the diagnosis of YOAD and YOFTD (Table 2). PPV was very low but the NPV 100%, with low likelihood ratios of 0.14 to 0.72, suggesting that a normal EEG makes the diagnosis of YOAD or YOFTD very unlikely. Slow wave changes and epileptiform activity increase the probability of YOAD but not YOFTD (Table 2).

The presenting problem data is shown in Table 5. The YOAD group was significantly more likely to present with memory deficits than the YOFTD group (100% vs. 67.9%, *P* < 0.00001). The YOFTD group was significantly more likely to present with behavioural changes compared to the YOAD group (64.3% vs. 13.6%, χ^2^ 30.6, *P* < 0.00001). There was no significant difference between the two groups in terms of presentation with mood disturbance (25.2% vs. 26.9%, χ^2^ 0.0007, *P* = 0.98), language deficits (13.6% vs. 21.4%, χ^2^ 1.05, *P* = 0.31), or motor disturbances (3.9% vs. 3.6%, χ^2^ 0.006, *P* = 0.94). Bilateral frontal and temporal epileptiform activity was associated with memory and mood disturbance in YOAD and memory and behavioural change in YOFTD.

**Table 5:** The number of patients with young onset Alzheimer’s disease or young onset frontotemporal dementia with specific presenting problems who had EEG abnormalities

EEG abnormality	Memory disturbance^a^	Behavioural changes^b^	Mood disturbance^c^	Language deficits^d^	Motor disturbance^e^
**Young onset Alzheimer’s disease**					
Total (*n* = 103)	103	14	26	14	4
Epileptiform activity only (*n* = 2)	2	0	1	0	0
Slow wave changes only (*n* = 51)	51	10	13	9	2
Both (*n* = 38)	38	4	9	5	2
Neither (*n* = 12)	12	0	3	0	0
**Young onset frontotemporal dementia**					
Total (*n* = 28)	19	18	7	6	1
Epileptiform activity only (*n* = 3)	3	3	0	0	0
Slow wave changes only (*n* = 7)	2	4	3	2	1
Both (*n* = 5)	5	1	2	1	0
Neither (*n* = 13)	9	10	2	3	0

^a^ Memory disturbance is defined as a change in any type of memory, such as short-term memory and long-term memory

^b^ Behavioural changes are defined as any change in behaviour such as an increase in aggression, inappropriate behaviours, or obsessionality

^c^ Mood disturbance is defined as a significant change in baseline mood that is not otherwise explained

^d^ Language deficits are defined as a decrease in language fluency, either with language production or language comprehension. Note that word finding difficulties are considered a memory disturbance

^e^ Motor disturbance is defined as a disturbance in motor functioning such as dystonia, apraxia, and paraparesis

Of the 131 patients with either YOAD or YOFTD, 12 patients developed seizures (9.2%) over 23 years of follow-up (Table 6). Patients who developed seizures were investigated with MRI scanning with contrast, blood tests including autoantibodies and search for antibodies to NMDA and other neuronal receptors were normal or negative in all 12 patients.

**Table 6:** Patients with clinical seizures and young onset dementia

Patient Number	Young onset dementia subtype	Onset of seizures	Type(s) of seizure	Patient’s EEG abnormalities
1	Alzheimer’s disease	1 year before onset of dementia	Focal unaware, myoclonic jerks	Bitemporal epileptiform activity, widespread^a^ slow wave changes
2	Alzheimer’s disease	3 years before onset of dementia	Generalised tonic-clonic	Bitemporal epileptiform activity, widespread slow wave changes
3	Alzheimer’s disease	1 year after onset of dementia	Focal unaware	Right frontal epileptiform activity, widespread slow wave changes
4	Alzheimer’s disease	5 years after onset of dementia	Generalised tonic-clonic, myoclonic jerks	Epileptiform activity and slow wave changes in left hemisphere
5	Alzheimer’s disease	3 years before onset of dementia	Generalised tonic-clonic	Bitemporal epileptiform activity, widespread slow wave changes
6	Alzheimer’s disease	2 years after onset of dementia	Generalised tonic-clonic	Generalised^b^ epileptiform activity, generalised slow wave changes
7	Alzheimer’s disease	10 years before onset of dementia	Generalised tonic-clonic	Widespread epileptiform activity, widespread slow wave changes
8	Alzheimer’s disease	5 years before onset of dementia	Focal unaware	Widespread epileptiform activity, widespread slow wave changes
9	Alzheimer’s disease	3 years before onset of dementia	Focal unaware	Widespread epileptiform activity, widespread slow wave changes
10	Alzheimer’s disease	20 years before onset of dementia	Focal unaware	Widespread epileptiform activity, widespread slow wave changes
11	Alzheimer’s disease	5 years after onset of dementia	Generalised tonic-clonic, myoclonic jerks	No epileptiform activity, widespread slow wave changes
12	Frontotemporal dementia	10 years before onset of dementia	Focal unaware	Epileptiform activity in left hemisphere, no slow wave changes

^a^ Widespread abnormalities are defined as abnormalities seen in five or more discrete locations

^b^ Generalised abnormalities are defined as abnormalities that are seen throughout the brain without a discrete location

At the time of entry into the study and prior to the EEG, no patients were on cholinesterase inhibitors and anticonvulsants. None were on psychoactive drugs. Of the YOAD patients 10/103 were on escitalopram, as were 2/28 of the YOFTD patients at time of enrolment. All the YOAD patients were on cholinesterase inhibitors at the time of their seizures; adversely those patients who developed seizures prior to onset of YOD were not on cholinesterase inhibitors or psychoactive drugs. Eleven of these patients had YOAD (10.7% of the disease group), and one had YOFTD (3.6% of the disease group). Eight patients (66.7%) had seizures prior to the onset of their dementia (AD: median 3y, mode 3y, range 18y, max 20y, min 1y, IQR 7; one patient with FTD had a focal unaware seizure 10y before the onset of dementia); while four (33.3%) developed seizures later (median 3.5y, mode 5y, range 4y, min 1y, max 5y, IQR 3.5). Focal unaware seizures and generalised tonic-clonic seizures were the most common, with six patients (50%) having each kind of seizure. Myoclonic jerks developed in 3/11 patients with YOAD (27.3%). All patients with seizures and young onset dementia had EEG abnormalities, with 11/12 having epileptiform activity (91.7%).

## Discussion

In this prospective study, abnormal EEGs were noted in 88.3% of patients with YOAD, 53.6% of patients with YOFTD, and 4% of the healthy controls. This finding that patients with YOD are more likely to have abnormal EEGs is consistent with past studies [14, 27, 28]. Furthermore, that patients with YOAD are more likely to have EEG abnormalities than those with YOFTD is consistent with past research by Pijnenburg et al. [30]. Overall, this data suggests that the presence of abnormalities on EEG could be diagnostically suggestive of young onset dementia, and is more suggestive of Alzheimer’s disease than frontotemporal dementia. There was no association with APoE 4 alleles in both YOAD and YOFTD populations, as there was a high frequency of APoE 4 alleles in both [50]. Furthermore, the MRI and FDG-PET scans were diagnostic of AD and FTD. Therefore, there was no particular correlations with biomarkers, other than their diagnostic value.

Patients in the YOAD group were approximately twice as likely than patients in the YOFTD group to have slow wave changes on their EEG, which is a finding that agrees with other studies [30]. Furthermore, patients with YOAD were more likely to have generalised slow wave changes on EEG, with 47.2% having generalised changes compared to 0% in the YOFTD group. The widespread slow wave changes and reduced fast rhythms are thought to result from extensive functional disruption and dysregulation between cortical networks from neuronal death, especially between frontoparietal and frontotemporal areas. Axonal damage, synapse injury from toxic Aβ and tau peptides, and cholinergic deficits are not found in FTD [51–54]. These results suggest that the EEG may have a role in differentiating between the two conditions, which may be of use when there is diagnostic uncertainty.

No significant difference was found between the two groups in terms of the rates of epileptiform activity (YOAD 38.8% vs. YOFTD 28.6%), the proportion of specific types of epileptiform activity, or the locations of epileptiform activity. This fact may be explained by tau, which is a protein which has significant implications in the pathophysiology of both Alzheimer’s disease [55] and frontotemporal dementia [56]. Animal models have suggested a possible mechanistic role of abnormal tau proteins and tauopathies in epileptogenesis [57, 58]. This theory could also explain why both disease groups had rates of epileptiform activity that was far greater than the rates in the general population, which is estimated to be between 1 and 5% [59, 60]. Although the difference is insignificant in the present study, the slight increase in epileptiform activity in the YOAD group compared to the YOFTD group may have been due to the presence of Aβ deposits which may act as a trigger, as has been suggested in an animal model [20].

There were significant differences between the two groups in terms of their presenting problems, with the YOAD group more likely to present initially with memory deficits and the YOFTD group more likely to present with behavioural changes. This was an expected result, as Alzheimer’s disease typically begins as a decline in memory, and frontotemporal dementia as a change in behaviour or personality [61]. There were no significant differences in terms of rates of mood disturbance, language deficits, or motor dysfunction at presentation. There was no significant correlation between any of the EEG abnormalities and presenting problems.

Within the two disease groups, eight patients developed seizures prior to dementia diagnosis, while 4 patients after. 3.1% of the cohort with dementia developed seizures after diagnosis, which is far greater than the approximate incidence for epilepsy and first seizures in the total population [62]. This finding is in keeping with past studies [40, 63, 64]. Furthermore, the brain changes associated with Alzheimer’s disease are thought to begin 10–20 years before the disease manifests with symptoms [65, 66], which could explain the 6.1% of the cohort who had a seizure history preceding the onset of their dementia. The seizure prevalence figures from the present study may be underestimated, as another study found that most seizures in Alzheimer’s disease were non-convulsive and may be unrecognised [32].

The prospective design of this study is a considerable strength, as it eliminates potential sources of significant bias present in previously published retrospective investigations. Furthermore, the comprehensive design, with inclusion of each patient’s presenting problem, is another strength as it helps set this data within the clinical context. Despite this, there are some limitations to the study. A larger sample size in the YOFTD group would have been beneficial, as would have digitalized EEG analysis. Each participant’s EEG was reported individually and separately with a single report being issued. Only one neurologist reported on all the EEGs after review and discussion of each EEG with the same technical staff who performed the studies, minimizing inter-rater invalidity.

## Conclusions

In conclusion, we propose that the EEG has a role as a diagnostic biomarker in young onset dementia. The presence of abnormalities, specifically slow wave changes—either focal or generalised—is more suggestive of YOAD than YOFTD. Furthermore, 9.2% of YOD patients had seizures, highlighting the clinical importance of the development of seizures prior to or after the onset of YOD; a finding which has important implications for patient management. In practical terms, a normal EEG makes the possibility of YOD unlikely. Moreover, the development of seizures in midlife might indicate a neurodegenerative aetiology.

12/131 patients with YOD developed seizures years before or after onset of dementia, 7/11 (63.6%) prior to the beginning of their dementia, and 4/11 (36.3%) after onset. In the absence of all other causes of seizures, and given the decades of follow-up for which no other causes for seizures have been identified, despite repeated MRI scans and blood investigations—including tests for autoantibodies and tests for anti-NMDA receptor and other related antibodies—we posit that the onset of seizures in mid-life might reflect an underlying neurodegenerative process in the absence of other causes. The prevalence of epilepsy is 1.1% at 60 years of age, with stroke and neurodegenerative disorders accounting for at least half [67]. In our population of 131 dementia subjects, 12 developed seizures representing a frequency of 9.2% greater than the predicted population prevalence of 1.1%, suggesting an association between seizures and dementia.

## Data Availability

The data that support the findings of this study are available on reasonable request from the corresponding author. The data are not publicly available as they contain information which could compromise the privacy of the participants of the study.

## List of Abbreviations

ACE-R: Addenbrooke’s Cognitive Examination – Revised 2005
ADRDA: Alzheimer’s Disease and Related Disorders Association
CSF: Cerebrospinal fluid
DASS: Depression, Anxiety and Stress Scores
EEG: Electroencephalography
IRDA: Intermittent rhythmic delta
MMSE: Mini-Mental State Exam
MRI: Magnetic resonance imaging
NINCDS: National Institute of Neurological and Communicative Disorders and Stroke
NPV: Negative predictive values
PET: Positron emission tomography
PPV: Positive predictive values
TFC: Total Function Capacity
YOAD: Young onset Alzheimer’s disease
YOD: Young onset dementia
YOFTD: Young onset frontotemporal dementia
